# Sociocultural Tailoring of a Healthy Lifestyle Intervention to Reduce Cardiovascular Disease and Type 2 Diabetes Risk Among Latinos

**DOI:** 10.5888/pcd10.130137

**Published:** 2013-11-27

**Authors:** Gia Mudd-Martin, Maria C. Martinez, Mary Kay Rayens, Yevgeniya Gokun, Janet C. Meininger

**Affiliations:** Author Affiliations: Maria C. Martinez, La Casita Center, Louisville, Kentucky; Mary Kay Rayens, Yevgeniya Gokun, University of Kentucky, Lexington, Kentucky; Janet C. Meininger, University of Texas, Houston, Texas.

## Abstract

**Background:**

Suboptimal lifestyle factors in combination with genetic susceptibility contribute to cardiovascular disease and type 2 diabetes risk among Latinos. We describe a community–academic collaboration that developed and explored the feasibility of implementing a socioculturally tailored, healthy lifestyle intervention integrating genomics and family history education to reduce risk of cardiovascular disease and type 2 diabetes among Latinos.

**Community Context:**

The community-based participatory research was conducted with communities in Kentucky, which has a rapidly growing Latino population. This growth underscores the need for socioculturally appropriate health resources.

**Methods:**

*Su Corazon, Su Vida* (Your Heart, Your Life) is a Spanish-language, healthy lifestyle educational program to reduce cardiovascular disease and type 2 diabetes risk among Latinos. Twenty natural leaders from an urban Latino community in Kentucky participated in sociocultural tailoring of the program and development of a genomics and family history module. The tailored program was presented to 22 participants to explore implementation feasibility and assess appropriateness for community use. Preintervention and postintervention assessments of genomic knowledge and lifestyle behaviors and qualitative postintervention evaluations were conducted.

**Outcomes:**

Postintervention improvements in health-promoting lifestyle choices and genomic knowledge specific to cardiovascular disease and type 2 diabetes suggested that the program may be effective in reducing risk. Feedback indicated the program was socioculturally acceptable and responsive to community needs.

**Interpretation:**

These findings indicated that a tailored healthy lifestyle program integrating genomics and family history education was socioculturally appropriate and may feasibly be implemented to reduce cardiovascular disease and type 2 diabetes risk in a Latino community with limited health care resources. The project highlights contributions of community-based processes in tailoring interventions that are appropriate for community contexts.

## Background

Cardiovascular disease (CVD) and type 2 diabetes mellitus (DM) are leading causes of illness and death among US Latinos. In the United States in 2010, 13.2% of Latinos aged 18 or older had DM (1). Rates of CVD, although lower among Latinos than in other population groups, are projected to increase as the Latino population ages (2). The public health implications are profound, given that Latinos represent 16.7% of the US population (3).

In combination with genetic susceptibility, suboptimal lifestyle factors including physical inactivity, unhealthful diet, and obesity contribute to CVD and DM risk among Latinos (4,5). Family history reflects genetic and lifestyle influences on chronic diseases, and family history information can be used to tailor prevention interventions. However, effective use of family history information may be hampered by community-level deficits in genomic knowledge (6).

Interventions facilitated by *promotores,* laypersons who understand local health issues and are dedicated to improving their communities’ health (7), have effectively promoted healthy lifestyles among Latinos. *Su Coraz*ó*n, Su Vida* (SCSV) is a *promotores*-facilitated educational program to reduce CVD risk among Latinos through healthy lifestyle promotion (8,9) that has been successfully adapted for DM prevention (10). Genomics and family history can support risk identification and motivate healthy behaviors (11). Integration of genomic and family history education into the SCSV program may further enhance effectiveness by supporting risk awareness and engagement in risk-reducing behaviors.

Effective intervention using SCSV has been demonstrated in US communities with a longtime Latino presence but not in “nontraditional” states where rapid growth of the Latino population has been more recent (5) and where culturally and linguistically appropriate health resources are limited. We describe a community–academic collaboration established in response to health needs of Latino residents in a nontraditional state. We describe the participatory process of tailoring the SCSV program and incorporating genomic and family history education, present results of an exploratory examination of sociocultural acceptability, and discuss the outcomes and public health implications.

## Context

The Latino population of Kentucky, estimated to be 140,000 (3.2% of the state’s population), more than doubled in size between 2000 and 2010, ranking fourth in the rate of growth in US states after South Carolina, Alabama, and Tennessee (12). Similar to the population nationally, most Kentucky Latino residents are from Mexico (61.3%) or other Latin American countries (22%); a smaller percentage are of Puerto Rican (6.4%) and Cuban (6.4%) origin (13). As a nontraditional Latino state, Kentucky has limited culturally and linguistically appropriate health resources (14). Resource limitations in combination with socioeconomic disparities negatively affect the health of Latino residents. More than one-third of Latinos in Kentucky live in poverty. The median annual income of people aged 16 or older is $18,000, and 16% are unemployed; 38% of the population is uninsured (15). The high cost of health care, lack of health care access for the uninsured, and lack of available linguistically and culturally appropriate health resources have been identified as major health-related barriers in surveys and focus group discussions with Latino residents of Louisville and Lexington, the 2 largest Kentucky cities, and are associated with poor health outcomes (16–18).

To address health needs of Kentucky’s Latino residents, a community–academic collaboration was established in 2008. The partnership originated from a longstanding relationship between the principal investigator (PI; G. M.) at the University of Kentucky and community leaders of the Latina Women’s Movement and La Casita Center in Louisville, Kentucky. The Latina Women’s Movement is a grassroots initiative dedicated to improving women’s well-being through programs that honor Latin American cultural traditions. La Casita Center is a community-based organization that provides services supporting Latino community development. Guided by principles of community-based participatory research (CBPR), the collaboration emerged from a mutually recognized need for a sustainable health promotion initiative, and a research team was formed that included the PI, the director of La Casita Center (who served as co-investigator; M. C. M.), and the founder of the Latina Women’s Movement (who was the project director).

**Figure 1:**
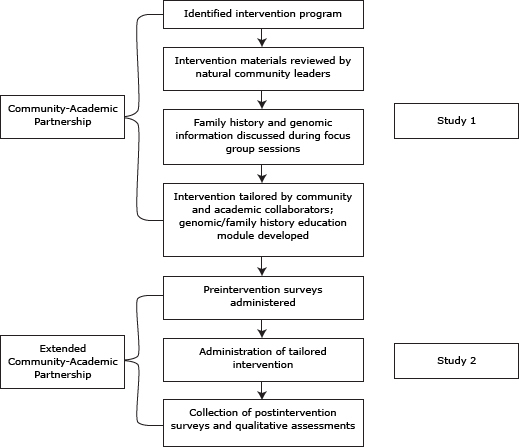
Flowchart presenting an overview of the process of the formative study (Study 1) and assessment of the intervention program (Study 2), *Su Corazon, Su Vida,* Kentucky, 2008–2010.

The team prioritized CVD and DM prevention through a health-promoting lifestyle intervention because of the high rates of these diseases among Latinos and the potential for the intervention to improve health outcomes in an environment with limited health care resources. Collaborating partners agreed that a *promotores*-facilitated program would have greatest potential for sustainability with long-term health effects. Specific objectives of the collaboration were to 1) identify and tailor a healthy lifestyle intervention to reduce CVD and DM risk for use among Latino residents in urban Kentucky and 2) explore the feasibility of implementing the community intervention (Figure 1).

## Methods

We conducted an extensive literature search to identify existing lifestyle programs with demonstrated effectiveness among Latinos. Team consensus was that the SCSV program could be tailored to address community needs and, as a *promotores*-facilitated intervention, had the greatest potential for sustainability. The program consisted of 8 educational sessions and a ninth review/evaluation session. Program details have been described previously (8).

To guide sociocultural tailoring of the SCSV program, formative research was conducted from 2008 through 2009 (Study 1). This research was followed by exploratory examination of implementation feasibility and acceptability of the tailored curriculum conducted during 2010 (Study 2). Both studies were approved by the University of Kentucky institutional review board.

### Formative research (Study 1)

Community partners were particularly interested in focusing on Latina women, whom they identified as more influential on family and community health behaviors than Latino men. It was decided to specifically engage Latinas in the sociocultural tailoring of the SCSV modules. Three *promotores* training sessions were also developed by community collaborators to complement the SCSV educational modules.

Twenty Spanish-speaking Latinas identified by community partners as natural leaders participated. Natural leaders were defined as women who were representative of area Latinas, involved in community activities, and well respected by community members. Before study initiation, participants gave written informed consent.

The 3 *promotores* training sessions and 8 SCSV educational modules were presented in Spanish during 11 two-hour sessions, with 1 module presented weekly. Participants provided feedback on the comprehensibility and sociocultural appropriateness of the modules. Each also participated in 1 of 4 focus group sessions conducted to guide the integration of culturally relevant family history and genomics education into the program. A final study session included summary review of the SCSV program with in-depth discussion of the potential value for promoting health in the community. All sessions were facilitated by the project director. To acknowledge their collaboration, participants received a gift card for sessions attended; childcare was also provided. The research team met regularly to review participant feedback and responses to modules. The iterative process of program tailoring was guided by individual and group feedback.

Overall evaluation of the program was positive. Participants valued education about the physiologic bases of the diseases but were most interested in learning about risk factors and risk reduction. To enhance self-efficacy in reducing risk, participants suggested that specific, achievable health-promoting activities be identified early in the program and emphasized throughout. The women recommended omitting optional program activities such as *fotonovelas* (supplemental short illustrated stories) to allow time to discuss practical applications of information. For example, participants identified and shared healthy recipes for commonly enjoyed foods and suggested time be provided during each session for such activities. Including stress reduction techniques and using educational models to support learning were also suggested.

Program adaptations in response to feedback included integration of time into each session for participants to identify and discuss context-specific health-promotion strategies. A psychiatric nurse practitioner with stress management expertise assisted with development of stress reduction techniques, including deep breathing and imagery exercises. Educational models complementing program modules were purchased, including a manipulable heart model and a visual display of sugar and salt content of foods commonly consumed in the Latino community.

Feedback from focus group sessions supported the value of integrating genomics and family history education into the SCSV program. Further details on the focus groups have been previously reported (19). Participants indicated that understanding of family health history and its implications for personal health is limited, and, although they expressed interest in learning about this topic, they also conveyed the importance of reinforcing that a heritable predisposition can be modified through healthy behaviors. This information supported the development of a genomics and family history module that includes basic education about genes and family history with implications for health and coinciding risk-reduction activities. Strategies for communicating about health history with family members were also provided.

There was enthusiastic consensus that the *promotores*-facilitated SCSV program could be successfully implemented in the community to improve health outcomes. However, participants suggested that both men and women be included in an educational initiative. Although there was agreement that women strongly influence family health behaviors, participants believed the support of male family members is vital to successful behavior change.

To maintain fidelity to the SCSV program, we adhered to the original implementation framework and retained key educational components. The genomics and family history module was structured to complement SCSV, using a presentation format identical to that of other program modules. The *promotores* training modules were not included in the final tailored program but were maintained as a separate training protocol for community development. An outline of the adapted SCSV modules and concepts covered is provided in Table 1.

**Table 1:** Adapted Modules and Key Concepts for the Modified *Su Corazon, Su Vida* (Your Heart, Your Life) Program, Kentucky, 2008–2010^a^

Session No.	*Sesiones de Su Corazón Su Vida* (Your Heart, Your Life Sessions)	Concepts Presented in Each Session
1	¿*Está usted en riesgo de desarrollar enfermedades del corazón*? (Are you at risk for heart disease or type 2 diabetes?)	General health, cardiovascular health and diabetes Cardiovascular disease risk factors Cardiovascular disease prevention and health promotion Recognizing and responding to symptoms of a heart attack and stroke
2	*Dígale SÍ a la actividad física* (Say YES to physical activity)	Health benefits of physical activity Types and amount of time to engage in physical activity Ways to be more physically active throughout the day
3	*Controle su presión arterial alta* (Control your high blood pressure)	Blood pressure: what is it Blood pressure screening and interpretation Prevention of hypertension and health-promoting behaviors Self-management of hypertension
4	*Conozca el historial de la salud de la familia* (Know your family history)	Genes: what are they and what is their role in health and disease How genes are transmitted in families What is family history Implications of family history for personal and familial health relevant to cardiovascular disease and type 2 diabetes Communicating with family members about health history
5	*Prevenga la diabetes tipo 2* (Prevent type 2 diabetes)	Diabetes: what is it, with focus on type 2 diabetes Screening and understanding blood glucose levels Prevention of type 2 diabetes and health promoting behaviors Self-management of type 2 diabetes
6	*Mantenga un peso saludable* (Maintain a healthy weight)	Overweight/obesity and risk for cardiovascular disease and type 2 diabetes Ranges of healthy and unhealthy weight Weight management and health promoting behaviors
7	*Coma de una manera saludable* (Eat healthfully)	Healthy eating to reduce cardiovascular disease and type 2 diabetes risk Reducing dietary intake of sugar, salt, and cholesterol Reading and understanding food labels Planning and preparing heart healthy meals Low-cost healthy foods and meals
8	*Goce de la vida sin el cigarrillo* (Enjoy life without cigarettes)	The effects of cigarettes and secondhand smoke on health Smoking cessation strategies Stress reduction (stress reduction techniques were integrated into other sessions; more in-depth education was included in this module with practice of techniques)

a Adapted from the *Su Corazon, Su Vida* manual (8).

### Exploration of implementation feasibility and sociocultural acceptability (Study 2)

For this study, we reached out to North Central Area Health Education Center (NC-AHEC), an organization with an extensive history of providing community education through a well-established *promotores* program. An uncontrolled preintervention/postintervention was designed with the following specific aims:

1. Determine the feasibility of implementing the tailored SCSV program;
2. Examine preliminary indicators of the effect of the program on healthy lifestyle choices and genomic knowledge related to CVD and DM;
3. Gather evaluative feedback on sociocultural acceptability of the program.

#### Sample and setting

This study was conducted in Lexington, Kentucky. Guided by participant feedback during the formative research, we decided to recruit both men and women. Perspectives of *promotores* and lay community members were important for evaluating program acceptability. We therefore extended verbal invitations to participate to 40 *promotores* with previous training from NC-AHEC. Lay community members were recruited through flyers posted at sites frequented by Latino community members. Inclusion criteria were being a Latino aged 18 years or older whose primary language was Spanish.

Two intervention groups were conducted: one at the NC-AHEC office and the other at a local library used for multiple community activities. Each site is easily accessible and familiar to Latino community members.

#### Measures

Via a written survey, we assessed demographic characteristics by using individual items. Genomic knowledge of CVD and DM was measured using a 29-item Spanish-language instrument with true/false response options. Genomic knowledge refers to understanding disease risk in relation to genes, family history, and lifestyle behaviors. The instrument included 5 items related to genetic predisposition, 8 to family history, 9 to behavioral risk, and 7 to genetic and behavioral risk factors (Table 2). The total knowledge score was the number of correct responses. Originally developed to assess genomic knowledge associated with DM (20), items relevant to CVD were added for this study. The Spanish-language version of the Health-Promoting Lifestyle Profile II (HPLP II) instrument (unpublished material, S.N. Walker, K. Sechrist, N. Pender, 1995) was used to assess healthy lifestyle choices. The scale comprises 52 items and 6 subscales; 4 response options range from 1 (“never”) to 4 (“routinely”). The total score for the instrument and the 6 subscale scores (health responsibility, physical activity, nutrition, spiritual growth, interpersonal relations, and stress management) were obtained by averaging all relevant items for each; higher scores indicated more healthful choices. Cronbach’s α for the full HPLP II scale was 0.92 for this sample.

**Table 2:** Genomic Knowledge of Cardiovascular Disease and Type 2 Diabetes, Instrument Sample Items, *Su Corazon, Su Vida* (Your Heart, Your Life) Program, Kentucky, 2008–2010

Concepts	Sample Items
Genes and cardiovascular disease	*¿Sólo los genes causan las enfermedades del corazón?* (Are genes the only cause of cardiovascular disease?)
Family history and cardiovascular disease	*¿Las enfermedades del corazón pueden heredarse dentro de una familia?* (Can heart disease run in families?)
Lifestyle behaviors and cardiovascular disease	*¿Hacer ejercicio ayuda a prevenir las enfermedades del corazón?* (Can exercising help to prevent heart disease?)
Genes and type 2 diabetes	*¿Los genes es uno de los factores que puede influir si una persona obtiene la diabetes tipo 2? *(Are genes one of the factors that can influence whether a person develops type 2 diabetes?)
Family history and type 2 diabetes	*¿Si una persona tiene familiares con diabetes tipo 2, está esta persona más propensa de obtener la diabetes tipo 2?* (Is a person with a family history of type 2 diabetes at greater risk to develop the disease?)
Lifestyle behaviors and type 2 diabetes	*¿Si una persona está propensa a obtener la diabetes tipo 2, puede disminuir el riesgo alimentándose sanamente?* (If a person is at risk to develop type 2 diabetes, can they reduce their risk by eating healthfully?)
Genes, lifestyle, and type 2 diabetes	*¿Por lo general, el desarrollo de la diabetes tipo 2 depende tanto del modo de vida como de los genes?* (In general, does the risk for developing type 2 diabetes depend as much on lifestyle as on genes?)

#### Procedures

A *promotora* with extensive experience conducting community-based health programs and with providing *promotores* training assisted the PI to collect baseline data. Educational modules were presented to each group in Spanish by the *promotora* during 8 weekly 2-hour sessions. The PI attended all sessions, taking field notes and evaluating protocol fidelity. A final session was used to collect postintervention data; feedback on the program was provided during an audiotaped discussion held with each group. Participants received a gift card and childcare was provided during each session.

#### Analysis

Comparisons of genomic knowledge and HPLP II scales and subscales from preintervention to postintervention were conducted using paired *t* tests. This analysis was completed using SAS version 9.3 (SAS Institute, Inc, Cary, North Carolina); an α level of .05 was used. Discussion session recordings were transcribed verbatim and transcriptions reviewed by the PI and *promotora* to identify salient themes.

## Consequences

### Sample description

Twenty-two participants, which included 1 group of 5 participants and a second group of 17, were enrolled and completed baseline assessments; 10 had previous *promotores* training. One participant attended all sessions but was unable to attend the final assessment; a second attended several sessions but a work schedule change precluded completing the intervention. Two participants did not attend after the first meeting. Eighteen participants (82%) completed the postintervention assessment. Of those who completed the assessment, 14 attended all 8 educational sessions; 8 attended 6 to 7 sessions.

The average age of participants in Study 2 was 44.5 years (standard deviation [SD] = 15.3 y). Men participated in each group but most participants were female (73%). Participants were from Mexico (59%) and South (36.4%) and Central (4.6%) America. Most participants were married (77%). More than half had some postsecondary education (52%); most were not employed (64%). Most reported their health to be “good,” “very good,” or “excellent,” the highest 3 ratings of a 5-level self-rated health assessment (68%). Most did not have health insurance (67%) or a health care provider (71%).

### Quantitative outcomes

**Figure 2:**
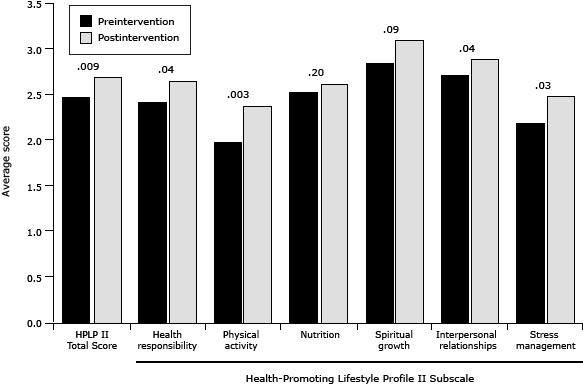
Average total and subscale scores preintervention and postintervention on the Health-Promoting Lifestyle Profile II. *P* values are shown at the top of columns. **Table:** 

There was a significant increase in genomic knowledge from preintervention (15.59, SD = 7.25) to postintervention (20.50, SD = 10.22; *P* = .03). The overall score on the HPLP II scale was 2.45 (SD = 0.37) at preintervention and 2.68 (SD = 0.37) at postintervention (*P* = .009). There was a significant increase in the HPLP II subscale scores for health responsibility (*P* = .04), physical activity (*P* = .003), interpersonal relations (*P* = .04), and stress management (*P* = .03); change over time was not significant for nutrition or spiritual growth (Figure 2).

Health-Promoting Lifestyle Profile II Subscale	Mean Score		*P* Value
	Preintervention	Postintervention	
**Total**	2.45	2.68	.009
**Health responsibility**	2.41	2.64	.04
**Physical activity**	1.97	2.35	.003
**Nutrition**	2.51	2.60	.20
**Spiritual growth**	2.84	3.10	.09
**Interpersonal relationships**	2.71	2.88	.04
**Stress management**	2.18	2.47	.03

### Qualitative feedback

Responses to program adaptations indicated that these were of benefit. Participants in both groups enthusiastically engaged in discussions on practical strategies for healthy lifestyles. During presentation of the genomic and family history module, participants expressed much interest in the topic. Several commented that they had heard of genes and associations with health but better understood this information after the presentation. Comments regarding positive experiences using relaxation techniques in response to daily stressors and appreciation of the educational models used to support learning indicated the benefit of each.

During the postintervention discussion, participants commonly expressed that the program had influenced their behaviors. Several shared that positive health effects, particularly weight loss, had occurred during the course of the program. Participants in both groups stated they were more aware of unhealthy eating habits. One participant said that she now read food labels when grocery shopping. Many talked about sharing educational materials and information with family members and stated that family members were engaging in more healthful behaviors as a result. More global comments on the program included positive feedback regarding the format and pace of the educational presentations.

A difficulty noted by several participants was that, although they were generally aware of the health status of first-degree relatives, they were less aware of that of their second-degree relatives. Several participants were hesitant to communicate with family members about health history because of privacy concerns. They thought family health discussions should occur naturally rather than information sought directly but agreed that increased family history awareness would encourage future discussions.

## Interpretation

Study results indicate that the socioculturally tailored SCSV program integrating genomic and family history education is feasible and appropriate to implement in a community setting among Latinos in a nontraditional area of residence. Outcomes also suggest that the program has potential to improve health behaviors and increase knowledge of genomics and family history of CVD and DM.

Program tailoring was guided by CBPR principles that included building on and extending community-academic relationships; mutual identification of community health needs and approaches to address these; and sociocultural tailoring, implementation, and evaluation of a risk-reduction program. Community partners were crucial to identifying health needs and guiding development of the research program. The academic component of the partnership was important in guiding scientific aspects of research. Participant collaborators in the formative research provided feedback critical for tailoring the SCSV program and informing development of the genomics and family history educational module. Strategizing with NC-AHEC representatives and the collaborative efforts of a *promotora* with the organization were essential to successful program implementation.

Community–academic collaboration foundational to CBPR presents both unique opportunities and challenges, both of which were experienced during this study. Effective communication among partners is vital to successful collaboration. In our study, collaborative processing, brainstorming, and problem-solving provided a solid foundation for relationship building and maintenance. Flexibility is also essential, as highlighted by study protocol changes guided by community partners. These resulted in improved outcomes but required significant cooperation and mutual engagement in the research process.

There were several study limitations. First, Study 1 was conducted with Latinas; inclusion of Latino males may have provided differences in program tailoring. However, outcomes from the exploratory study indicate the tailored intervention is appropriate for use with men and women. Second, although the small sample size for Study 2 was sufficient to indicate sociocultural appropriateness of the program, demonstrating effective CVD and DM risk reduction will necessitate conducting the intervention in a larger sample with more diverse representation of Latino countries of origin, a more rigorous research design, and a longer follow-up assessment period.

The outcomes of this study provide preliminary indication that the tailored healthy lifestyle intervention, including integration of genomic and family history education, is socioculturally appropriate and may provide a foundation for a sustainable program of health promotion for Latinos in a community with limited culturally and linguistically suitable health care resources. As important, the CBPR process used in program tailoring may provide guidance for addressing the health needs of Latino community members in other nontraditional areas of residence.
